# Corrosion Resistance of MgZn Alloy Covered by Chitosan-Based Coatings

**DOI:** 10.3390/ijms22158301

**Published:** 2021-08-02

**Authors:** Iryna Kozina, Halina Krawiec, Maria Starowicz, Magdalena Kawalec

**Affiliations:** Faculty of Foundry Engineering, AGH University of Science and Technology, Reymonta 23 Street, 30-059 Krakow, Poland; i.matviichuk7@gmail.com (I.K.); mariast@agh.edu.pl (M.S.); kawalec@agh.edu.pl (M.K.)

**Keywords:** chitosan coating, magnesium alloy, corrosion

## Abstract

Chitosan coatings are deposited on the surface of Mg20Zn magnesium alloy by means of the spin coating technique. Their structure was investigated using Fourier Transform Infrared Spectroscopy (FTIR) an X-ray photoelectron spectroscopy (XPS). The surface morphology of the magnesium alloy substrate and chitosan coatings was determined using Scanning Electron Microscope (FE-SEM) analysis. Corrosion tests (linear sweep voltamperometry and chronoamperometry) were performed on uncoated and coated magnesium alloy in the Hank’s solution. In both cases, the hydrogen evolution method was used to calculate the corrosion rate after 7-days immersion in the Hank’s solution at 37 °C. It was found that the corrosion rate is 3.2 mm/year and 1.2 mm/year for uncoated and coated substrates, respectively. High corrosion resistance of Mg20Zn alloy covered by multilayer coating (CaP coating + chitosan water glass) is caused by formation of CaSiO_3_ and Ca_3_(PO_4_)_2_ compounds on its surface.

## 1. Introduction

Because of low density and particularly good mechanical properties, magnesium alloys are widely used as a construction material in the automotive and aerospace sectors [1,2]. In recent years, a lot of attention has been paid to magnesium alloys as temporary biodegradable implants. Magnesium alloys are known to be biocompatible and nontoxic materials. The excess of magnesium ions can be safely excreted in the urine with ease, and Mg has positive effect on the growth of new bone tissue [3,4]. Moreover, the density and Young’s modulus of Mg alloys is closer to that of human bone compared to many other metallic materials used as implants [5].

The main obstacle for application of magnesium alloys as a temporary, biodegradable implant is their fast corrosion rate in the simulated body fluids (SBF). Therefore, it is necessary to use protective coatings that will slow down the corrosion rate. Different types of protective coatings were deposited on magnesium substrates. The most popular coatings are hydroxides-based coatings [6,7], metal oxide based coatings [8,9], silane sol-gel coatings [10,11,12], Ca-P coatings [13,14,15] and polymer coatings [16,17].

Some polymer coatings include chistosan. This compound is a natural biopolymer which exhibits particularly good biocompatibility, biodegradability, and antibacterial activity. Therefore, it found wide application in the medical sector [18,19,20]. Chitosan coatings can be deposited by dip coating [21,22,23], spin coating [3], layer–by–layer (LBL) coating (multilayer deposition) [24,25], chemical conversion [26], or aerosol deposition (AD) [27]. Chitosan is also widely used to prepare coatings on magnesium alloys by electrophoretic deposition (EPD) [28,29,30,31,32,33,34,35].

Coatings fabricated from the crosslinking of chitosan with genipin have beneficial influence on the corrosion resistance of AZ31 magnesium alloy in SBF [22,23]. It was shown [36,37] that chitosan coatings, with a crosslinking degree of up to 42%, provide good anticorrosive properties for AZ31 alloy in SBF. By contrast, chitosan coatings with a higher crosslinking degree are brittle are susceptible to the formation of cracks. Therefore, they are more prone to corrosion damage in SBF.

Some studies have revealed that multilayer chitosan-based coatings also have beneficial effects on the corrosion resistance of Mg alloys in SBF. These coatings were prepared on Mg-Zn-Ca alloy by micro arc oxidation (MAO) followed by dip-coating in chitosan solution. Pores present in the MAO coating were then sealed by chitosan. The presence of multilayer coatings causes a decrease of the corrosion current density by three orders of magnitude compared to that of bare Mg-Zn-Ca alloy [21].

Composite chitosan coatings were developed. They are usually prepared by addition of inorganic particles to chitosan suspension. They include bioactive glass (BG) [21,24,26,28,32,33,38,39], hydroxyapatite [27,29,34,35,40], carbon nanotubes [41], Fe_3_O_4_ nanoparticles [34,35], and mineralized bone allograft nanoparticles [42]. Chitosan/BG coatings reduce the corrosion rate of magnesium-based substrates in SBF [21,28,32,34]. Amorphous calcium and magnesium phosphate species were detected at the surface of chitosan/BG coated magnesium [26]. Those containing 0.4 g/L of BG significantly improve the corrosion resistance of AZ91 alloy in SBF, and they have a greater ability to form apatite compared to other groups [28].

Haise et al. [30] have shown that the addition of SiO_2_ particles to chitosan/BG coatings gives a topography favorable for the formation of hydroxyapatite when the coatings are in contact with SBF. Furthermore, the corrosion resistance of WE43 alloy was found to be increased with the application of chitosan/BG coatings containing SiO_2_. The addition of inorganic nanoparticles, such as hydroxyapatite (HA), titanium dioxide, and iron oxides, usually enhanced implant integration with host tissue, corrosion resistance, and has an antibacterial effect [27,34,35,40]. It was proved that the composite HA-BG-Fe_3_O_4_-chitosan coatings can effectively improve the hemocompatibility and the corrosion resistance of biodegradable magnesium alloy [34].

In the present paper, the corrosion behavior of Mg20Zn alloy, covered by chitosan-based coatings, is investigated in the Hank’s solution at 37 °C. Coatings were deposited using the spin coating method. This method is commonly used for deposition of polymer coatings on conductive, insulator, and semiconductor substrates. The main advantages are to produce fine, thin, and uniform polymer coatings. Moreover, the spin coating method is a relatively inexpensive compared to other deposition techniques. The influence of the structure of coatings (monolayer coating composed of chitosan with water glass (chitosan_WG) and multilayer coating composed of Ca-P as the first sublayer and chitosan_WG as the second sublayer) on the corrosion behavior and corrosion rate is quantified. This is performed using scanning electron microscopy (SEM) coupled with energy dispersive X-ray spectroscopy (EDS), Fourier-transform infrared (FTIR) spectroscopy, X-ray photoelectron spectrometry (XPS) and corrosion tests (linear sweep voltammetry (LSV), and open circuit potential (OCP) coupled with the hydrogen evolution method.

## 2. Materials and Methods

### 2.1. Materials

The Mg20Zn alloy was used as substrate for deposition of chitosan-based coatings. This alloy was selected because it does not contain toxic elements (zinc is a safe element for the human body). Moreover, the addition of zinc to magnesium alloy enhances its corrosion resistance in the simulated body fluids and in solutions containing a high amount of chloride ions. In the literature, there are many corrosion studies on magnesium alloys containing a few wt.% of zinc (<5 wt.%), but not on alloys richer in Zn. This alloy was delivered in the form of rods (diameter: 10 mm and length 1000 mm) by Goodfellow. Its chemical composition is: 20 wt.% Zn and 80 wt.% Mg. Rods were cut (height of 8 mm). The electrical contact to the lateral face of rods was made using an insulating cable. The rods (including the electrical contact) were embedded in an epoxy resin. The upper face of embedded rods (surface area in contact with electrolyte of 0.785 cm^2^) was ground with emery paper (1200 grit), cleaned by sonication for 60 s in ethanol, rinsed with ethanol, and then dried with argon.

### 2.2. Coatings Preparation

The first coating studied (noted “Ca-P coating”) was chemically formed by immersing the substrate (prepared as described in Section 2.1) in a solution containing 70 mL H_2_O, 30 mL C_2_H_5_OH, 2 g Ca(NO_3_)_2_•2H_2_O and 0.7 g NaH_2_PO_4_ for 12 h. The solution was stirred at 250 rpm.

The second coating (noted by “chitosan_WG coating”) was a chitosan-based coating deposited in a solution containing 50 mL of solution S1, 0.5 g Na_2_SiO_3_ and 1 mL of water glass (Chempur, SiO_2_/Na_2_O molar modulus 2.4–2.6). Solution S1 contains 2 g of chitosan (Acros Organics, molecular mass 100 kDa–300 kDa) dissolved in 100 mL of 1 vol.% lactic acid solution. To completely dissolve chitosan, S1 was stirred for 1 h at a temperature of 40 °C. This coating was prepared using the spin-coating method. The procedure used consists of four steps (step 1 for 5 s at 250 rpm, step 2 for 30 s at 1000 rpm, step 3 for 60 s at 1500 rpm, and step 4 for 60 s at 250 rpm). This procedure was repeated 6 times, resulting in a coating composed of six polymer sublayers.

The third coating studied is a multilayer coating (noted “Ca-P/chitosan_WG coating”). The Ca-P coating was first formed as described in the first paragraph of this section (first sub-layer). After this pretreatment, the “chitosan_WG coating” was deposited as described in the second paragraph (second sublayer).

### 2.3. Characterization Techniques

Top-view surface imaging of samples was carried out with SEM (JEOL, JSM-5500LV, 3-1-2 Musashino, Akishima, Tokyo 196-8558, Japan). An acceleration voltage of 20 kV was used. Intermetallic phases present in the Mg20Zn alloy were identified by XRD with Philips PW-3710 X’PERT diffractometer (Malvern Panalytical B. V. Lelywey 1, Almelo, 7602 EA, The Netherlands (formerly PANanalytical)) using Cu-Ka radiation.

FTIR analysis was realized on coated specimens with a Thermo-Scientific Nicolet 6700 spectrophotometer (Thermo Scientific, USA Thermo Fisher Scientific 168 Third Avenue, Waltham, MA 02451, USA) equipped with an attenuated total reflectance accessory (ATR). FTIR spectra were obtained over a scan range of 4000 to 500 cm^−1^.

XPS analysis was performed after deposition of chitosan-based coatings and after immersion of these coatings in the Hank’s solution for 110 h. The equipment is a SIA 100 Cameca Riber (CAMECA 29 Quai des Grésillons, 92622 Gennevilliers Cedex, France) apparatus and a nonmonochromated Al Ka line (energy of 1486.7 eV, power of 50 W and X-ray beam diameter of 200 microns). A Mac 2 semi-imaging spectrometer was used with a resolution of 1.3 eV (width of Ag 3d5/2 level). C(1s) peak from pollution (285 eV) was considered for the energy calibration. During XPS measurements, the residual pressure of the analysis chamber was maintained below 10^−7^ Pa. Spectra were treated with the CasaXPS 2.3.10 software package and ionization cross-sections from Landau were used to quantify the semi-empirical relative sensitivity factors.

### 2.4. Corrosion Measurements

Corrosion tests were carried out on uncoated and coated Mg20Zn substrates in the Hank’s solution. The chemical composition of the Hank’s solution is: 8 g/L NaCl, 0.4 g/L KCl, 0.140 g/L CaCl_2_, 0.350 g/L NaHCO_3_, 0.217 g/L NaH_2_PO_4_, 0.06 g/L Na_2_HPO_4_, 0.406 g/L MgCl_2_•6H_2_O, 0.029 g/L MgSO_4_, and 1 g/L D-glucose. Corrosion measurements were performed using a standard three-electrode cell. The reference electrode is an Ag/AgCl (3M KCl) reference electrode and the counter electrode is a Pt grid (4 × 5 cm^2^). All the experiments were performed with a Metrohm Autolab PGSTAT128 Potentiostat/Galvanostat and Nova 2.1 software.

Linear sweep voltammetry (LSV) curves were plotted from −50 mV vs. OCP to the anodic direction, at a potential scan rate of 1 mV s^−1^. Prior to LSV experiments, the open circuit potential (OCP) was measured for 30 min (to reach the steady state). Chronoaperometry tests were also performed on the uncoated and coated specimens, at a potential of −1.250 V vs. Ag/AgCl (3 M KCl) for 120 s.

The corrosion rate of Mg20Zn alloy and coated specimens was calculated based on measurements of the volume of released hydrogen. The specimens were immersed in the Hank’s solution for 110 h at 37 °C, and the amount of hydrogen gas evolved was measured using a burette placed above the specimen, according to the method described in [2,43]. This section may be divided by subheadings. It should provide a concise and precise description of the experimental results, their interpretation, as well as the experimental conclusions that can be drawn.

## 3. Results

### 3.1. Microstructure of Mg20Zn Alloy

**Figure 1:**
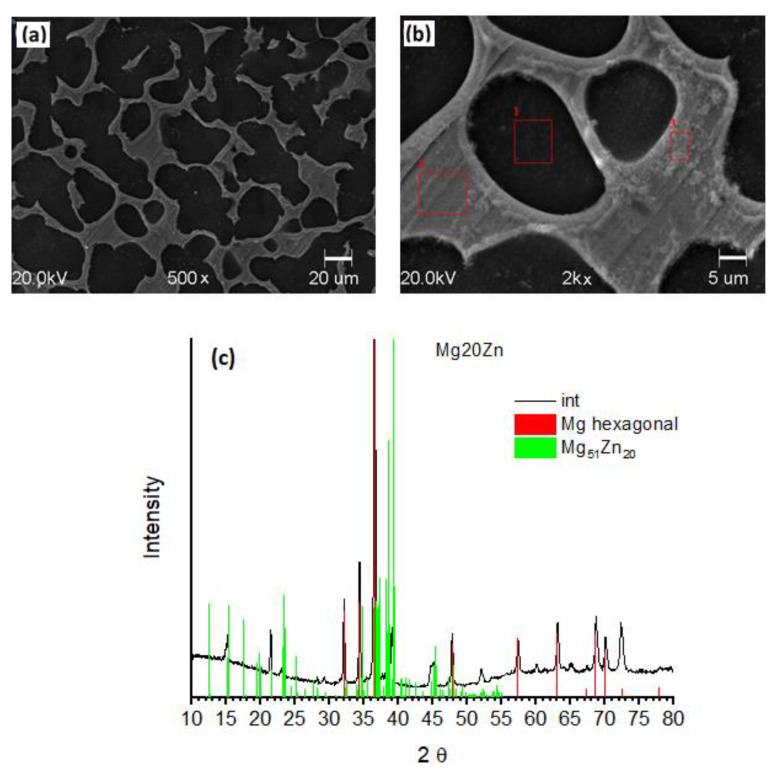
(**a**,**b**) SEM images of the Mg20Zn alloy after mechanical polishing. (**c**) XRD diffractogramm of the Mg20Zn alloy. Theoretical position of peaks related to α-Mg hexagonal (red bars) and Mg_51_Zn_20_ intermetallic (green bars) phases are reported.

To reveal the microstructure, specimens were ground with emery paper (1200 grit) and then smoothed with silica colloidal suspension. Mg20Zn alloy consists of two phases (Figure 1a,b), namely α-Mg hexagonal phase (matrix) and Mg_51_Zn_20_ intermetallic phase (located at grain boundaries of the matrix). The presence of these phases was confirmed by XRD, Figure 1c. EDS analysis was performed in sites containing the pure matrix (site 1 in Figure 1b) and the intermetallic phase (sites 2 and 3 in Figure 1b). The matrix is composed of magnesium (90.4 ± 2.7 at.%) with a small amount of zinc (2.5 ± 0.2 at.%) and oxygen (6.5 ± 1.5 at.%). The intermetallic phase contains magnesium (64.9 ± 6.5 at.%) and zinc (29.4 ± 5.2 at.%) with a small amount of oxygen (5.8 ± 1.2 at.%).

### 3.2. Microstructure of Chitosan-Based Coatings

**Figure 2:**
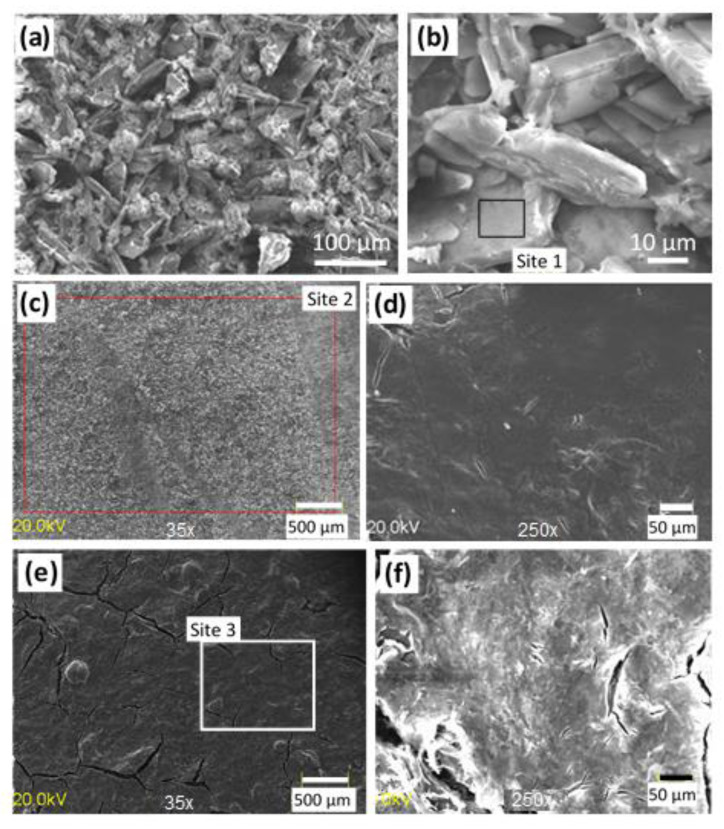
SEM images of (**a**,**b**) Ca-P (**c**,**d**) Ca-P/chitosan_WG and (**e**,**f**) chitosan_WG coatings deposited on the Mg20Zn alloy.

The Ca-P coating shows a rough surface with a crystalline structure (Figure 2a). At high magnification, a plate-like structure is visible, Figure 2b. Plates are mainly composed of calcium (64.6 at.% in site 1, Figure 2b) and phosphorous (25.2 at.%, site 1) with a small amount of oxygen (6.7 at.%, site 1).

The surface of the Ca-P/chitosan_WG coating appears smooth and compact, although some rare microcracks can be observed (Figure 2c,d). EDS analyses at low magnification (site 2 in Figure 2c) yields 35.3 at.% carbon, 45 at.% oxygen, 5.6 at.% silicon, and 2.3 at.% sodium (these elements come from the outer chitosan_WG sublayer), 6.1 at.% calcium and 5.4 at.% phosphorous. By contrast, the surface of the chitosan_WG coating deposited on the pure Mg20Zn alloy appears smooth, but numerous microcracks are observed, Figure 2e,f. EDS analysis performed on the surface of this coating (site 3 in Figure 2e) yields 55 at.% carbon, 37 at.% oxygen and 8 at.% silicon.

**Figure 3:**
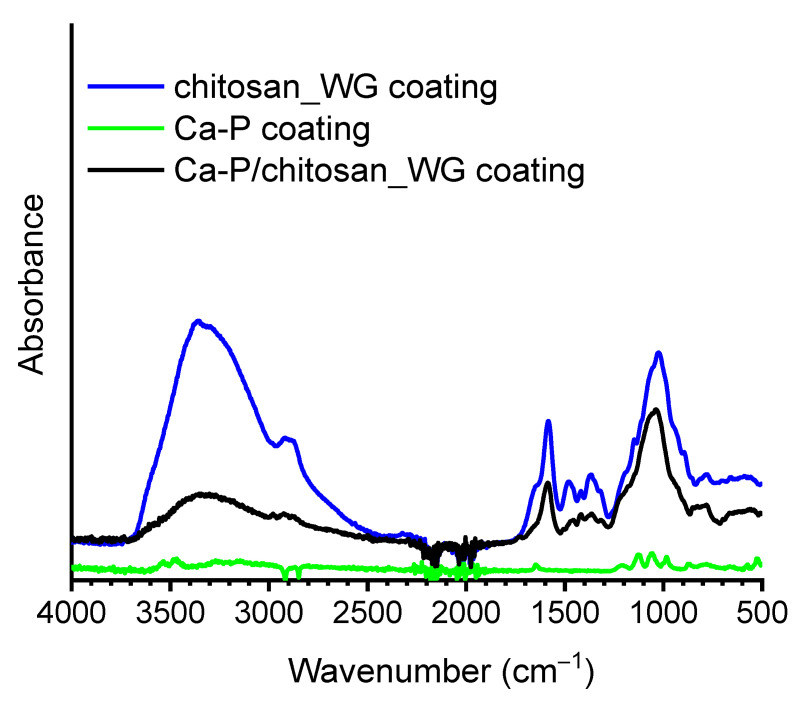
FT-IR spectra of the different coatings. The relevant bands are described in the text.

Figure 3 shows the FTIR spectrum of the chitosan_WG coating (blue curve). This FT-IR spectrum was compared to that of the chitosan coating (red curve in Figure 3). It can be noticed that the main band appearing in the spectrum of chitosan coating (red curve, Figure 3) is due to stretching vibration of hydroxyl (OH) and N-H groups in the range from 3700 cm^−1^ to 3000 cm^−1^ and C-H bond in −CH_2_ (v = 2930 cm^−1^), and −CH_3_ (v = 2875 cm^−1^) groups, respectively [29,44]. The band at 1560 cm^−1^ is assigned to –NH group with bending vibration, [42]. In addition, the bands revealed at 1376 cm^−1^ and 1061 cm^−1^ are related with the stretching vibration of C-O bound. The addition of water glass to chitosan results in a broadening of the band at 1023 cm^−1^. This is related to Si-O stretching vibrations in dimers [Si_2_O_7_]^6−^ and [SiO_3_]^2−^. A hump at 1640 cm^−1^ appears in the band between 1700 cm^−1^ and 1500 cm^−1^, and it originates from bending and stretching modes of OH groups. The shoulder at 880 cm^−1^ is associated with the asymmetric vibration isolated [SiO_4_]^4−^ groups [45,46,47]. The FTIR spectrum of the Ca-P/chitosan_WG is similar to that of the chitosan_WG coating, but all peaks have lower intensities. The FTIR spectrum obtained for the CaP coating (green curve in Figure 3) shows the adsorption bands in the range from 528 cm^−1^ to 1124 cm^−1^ assigned to phosphate groups [40].

**Figure 4:**
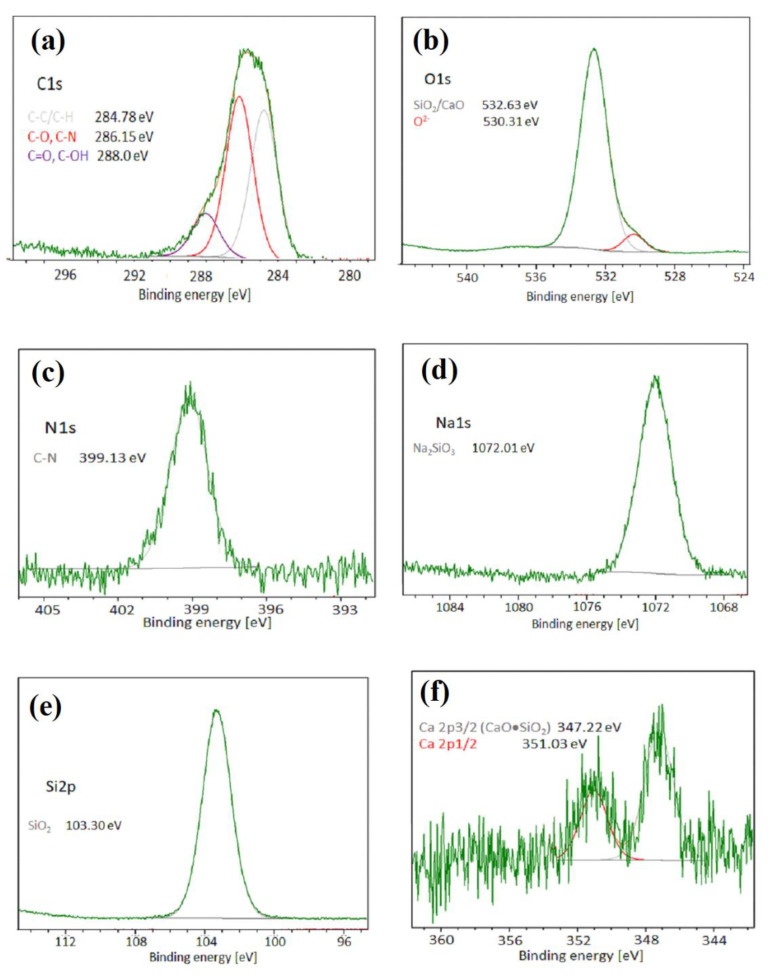
XPS spectra of the Ca-P/chitosan_WG coating deposited on the Mg20Zn alloy: (**a**) C1s, (**b**) O1s, (**c**) N1s, (**d**) Na1s, (**e**) Si2p, and (**f**) Ca2p levels.

XPS analysis was performed on the Ca-P/chitosan_WG coating. Quantitative analysis of XPS data shows that the chitosan_WG coating contains 18.2 at.% carbon, 1.7 at.% nitrogen, 53.3 at.% oxygen, 4.6 at.% sodium, and 22.0 at.% silicon. Figure 4a–f shows the C1s, O1s, Na1s, Si2p, Ca2p, and N1s XPS levels of the Ca-P/chitosan_WG coating. The C1s spectrum was deconvoluted into three contributions. The peaks at 284.78 eV, 286.15 eV, 288.0 eV are attributed to C-C/C-H, C-N, and C=O/C-OH chemical bonds, respectively. The peak at the binding energy 399.24 eV, recorded in the N1s spectrum, is assigned to C-N bond present in the chitosan molecule [48]. The peaks at the binding energy 530.31 eV and 532.6 eV are assignment to O^2−^ and SiO_2_/CaO, respectively [49]. One peak at the binding energy 1072.01 eV presents in the Na1s spectrum is attributed to Na_2_SiO_3_ [50]. The peak at the binding energy 103.3 eV present in the Si2p spectrum is assigned to SiO_2_ [49,51]. Figure 4f shows the typical bands 2p3/2 and 2p1/2 of the Ca2p orbital located at 347.2 and 351.03 eV, respectively. The peak at 347.2 eV corresponds to the Ca-O bond of CaSiO_3_ [52].

### 3.3. Corrosion Behaviour of Coated and Uncoated Mg20Zn Alloys under Potential Control

**Figure 5:**
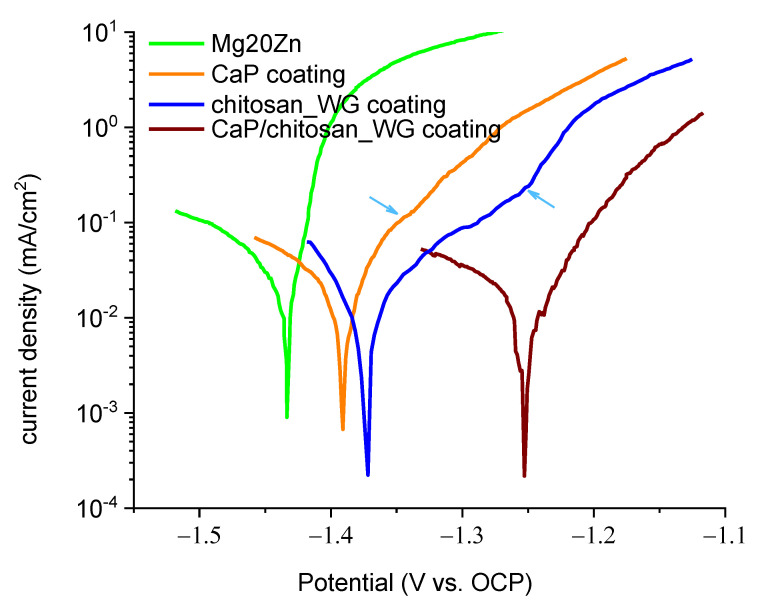
LSV curves (1 mV/s) of the Mg20Zn alloy and the different coatings in the Hank’s solution at 37 °C. Prior to LSV experiments, samples were immersed at the OCP for 30 min (OCP values: −1.47 V vs. Ag/AgCl for the alloy; −1.41 V vs. Ag/AgCl for Ca-P; −1.37 V vs. Ag/AgCl for Chitosan_WG, and −1.32 V vs. Ag/AgCl for CaP/chitosan_WG).

Figure 5 shows the polarization curves of the Mg20Zn alloy (substrate) and the substrate covered by the three different coatings. The current density in the anodic branch of the Mg20Zn substrate (green curve) increases sharply with increasing applied potential, exhibiting active behavior. The two monolayer coatings (Ca-P (orange curve) and chitosan_WG coatings (blue curve)) have very similar behaviors. In both cases, the current density in the anodic branch increases significantly, but the values are lower than those obtained on the Mg20Zn substrate. This shows that the presence of these two coatings has beneficial influence on the kinetics of anodic reactions. Note that the chitosan_WG coating behaves slightly better than the Ca-P coating (lower anodic current densities).

An inflection point is visible on both polarization curves, from which the current increases more rapidly (noted by arrows in Figure 5). This inflection point is more marked on the chitosan_WG coating (containing numerous cracks, Section 2) than on Ca-P (containing rare cracks). This can be related to the presence of cracks in the coatings providing new pathways for electrolyte penetration.

The presence of the multilayer coating (Ca-P/chitosan_WG coating) induces a sharp decrease of the current density in the anodic branch (wine curves, Figure 5), leading to a huge shift of the corrosion potential to the anodic direction (of about 0.2 V, with respect to the value obtained on the substrate). It can be underlined that the anodic current density measured at an applied potential of −1.2 V is 12.25 mA/cm^2^ on the Mg20Zn alloy, between 1 and 2.5 mA/cm^2^ on the two monolayer coatings, and only 0.05 mA/cm^2^ on the Ca-P/chitosan_WG coating. These results indicate that the specimen covered by the multilayer coating (Ca-P/chitosan_WG) has the highest corrosion resistance in the Hank’s solution and that the simultaneous presence of the two sublayers increase this resistance.

The corrosion potential of the three coatings is shifted to the anodic direction with respect to the corrosion potential of the substrate. This suggests that, in a coupling situation between the Mg20Zn alloy and the coatings (during a very long-term exposure to an aggressive environment that leads to a severe degradation of the coatings, for example), the coatings might act as a cathode (and the alloy as anode). This will be discussed in Section 3.5.

### 3.4. Hydrogen Evolution vs. Time under Free Corrosion (OCP)

**Figure 6:**
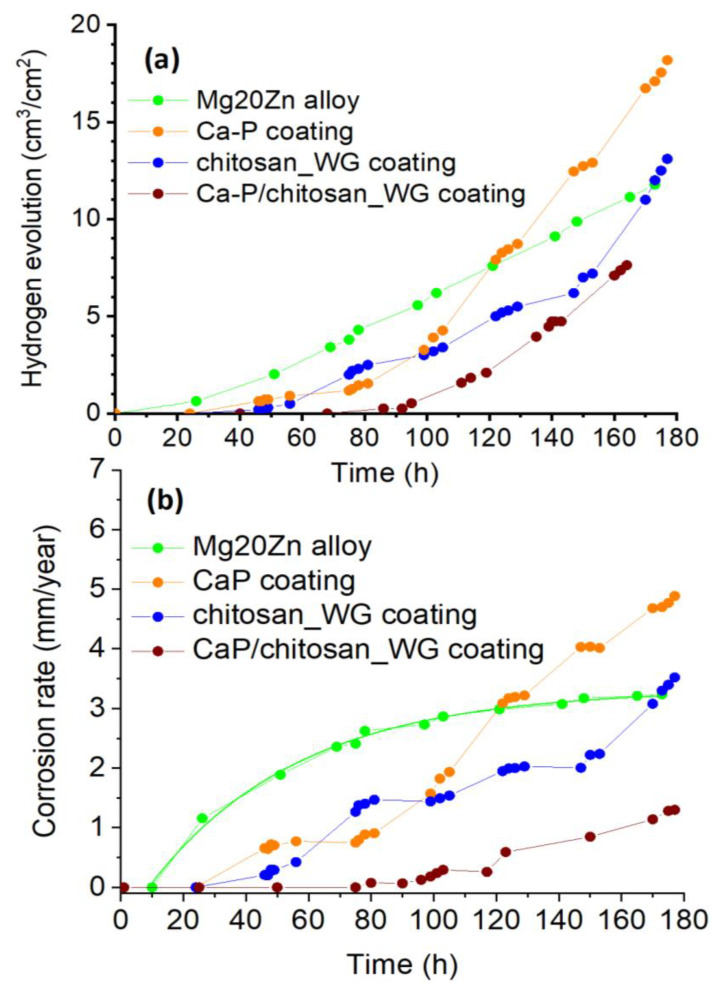
(**a**) Hydrogen evolution vs. time for the Mg20Zn alloy and the different coatings immersed at the OCP in the Hank’s solution at 37 °C. (**b**) Evolution of the calculated corrosion rate vs. time.

The hydrogen evolution method, described in reference [2,43], was used to evaluate the corrosion rate of the uncoated and coated specimens immersed at the open circuit potential (OCP) for 7 days in the Hank’s solution at 37 °C. Figure 6a shows the evolution of the released hydrogen volume versus time. As soon as the Mg20Zn substrate is immersed, the volume of released hydrogen increases linearly with time. This indicates that corrosion starts in the alloy just after immersion. The overall corrosion reaction of magnesium in aqueous solutions can be expressed by Reaction (1):

(1) $$\mathrm{Mg}+H_{2}O\to \mathrm{Mg}{\left(\mathrm{OH}\right)}_{2}+H_{2}$$

By contrast, hydrogen evolution starts to be observed from an immersion time of roughly 50 h for the two monolayer coatings (Ca-P and chitosan_WG coatings), indicating the onset of corrosion due to loss of protective properties of the coatings. Between 50 and 110 h of immersion, both coatings have similar behavior. From 110 h of immersion, the quantity of hydrogen released is significantly greater for the Ca-P coating than for the chitosan_WG coating, suggesting that the Ca-P coating degrades faster. These results confirm those derived from polarization curves. Both monolayer coatings have similar behavior (they improve the corrosion resistance of the alloy), but the chitosan_WG coating has slightly better corrosion resistance than the Ca-P coating. Hydrogen evolution was noticed on the multilayer coating from 90 h of immersion. In addition, the quantity of hydrogen released was found to be systematically smaller than in the three other cases (Mg20Zn alloy and the two monolayer coatings). This confirms that the multilayer coating has the highest corrosion resistance.

### 3.5. Corrosion Rate vs. Time under Free Corrosion (OCP)

According to the stoichiometry of Reaction (1), the evolution of 1 mol of hydrogen gas corresponds to the dissolution of 1 mol of magnesium. Therefore, the corrosion rate of magnesium can be calculated on the base of Reaction (1), knowing the volume of hydrogen evolved during the corrosion process. One mole of hydrogen gas in the normal conditions (T_0_ = 273 K and p_0_ = 101,325 Pa) takes up the volume *V*_0_ = 22.4 dm^3^. The corrosion tests shown in Figure 6a were carried out at the temperature of *T*_1_ = 310 K and the pressure of *p*_1_ = 101,325 Pa. Therefore, the volume *V*_1_ of one mol of hydrogen released during the corrosion tests was calculated under these conditions (*T*_1_, *p*_1_) using Equation (2).

(2) $$p_{0}V_{0}T_{0}=p_{1}V_{1}T_{1}$$

*V*_1_ was found to be equal to 25.4 dm^3^. The corrosion rate of the coated and uncoated specimens was then determined from Equation (3) [53,54].

(3) $$v=87.6\;\left(\frac{m\;\left[\mathrm{mg}\right]}{d\;\left[\frac{g}{{\mathrm{cm}}^{3}}\right]\cdot s\;\left[{\mathrm{cm}}^{2}\right]\cdot t\;\left[h\right]}\right)\left[\mathrm{mm}/\mathrm{year}\right]$$

Figure 6b shows the evolution of the corrosion rates vs. time. The corrosion rate of the substrate increases exponentially vs. time (according to Equation (4)), to reach a steady state at 3.2 mm/year.

(4) $$v_{corr,\;substrate}=3.2-4e^{\left(-0.02.Time\right)}$$

In the presence of the monolayer coatings (Ca-P and chitosan_WG coatings), the corrosion rate increases faster than in the case of the substrate. For long immersion times (greater than 120 h for the Ca-P coating and 170 h for the chitosan_WG coating), the corrosion rate is greater than values measured on the substrate. Severe degradation of the monolayer coatings occurs locally during immersion and, as discussed in Section 3.3, galvanic coupling may exist between the alloy and the coating which remained intact. Galvanic coupling promotes corrosion, leading to corrosion rates greater in the presence of these coatings. For the uncovered alloy, only Mg_51_Zn_20_ intermetallic phases act as a cathode.

It is remarkable to note that the corrosion rate measured on the multilayer coating increases slowly with increasing time, indicating that this coating has excellent protective properties against corrosion and that no galvanic coupling has yet developed between the multilayer coating and the alloy. By contrast to the monolayer coatings, the multilayer coating helps to protect the alloy against corrosion in the Hank’s solution.

After 173 h of immersion in the Hank’s solution at 37 °C, the corrosion rate is as follows: 4.7 mm/year for the Ca-P coating, 3.3 mm/year for the chitosan_WG coating, 3.2 mm/year for the Mg20Zn alloy, and only 1.2 mm/year for the multilayer coating (Ca-P/chitosan_WG coating).

### 3.6. Corrosion Products under Free Corrosion (OCP)

**Figure 7:**
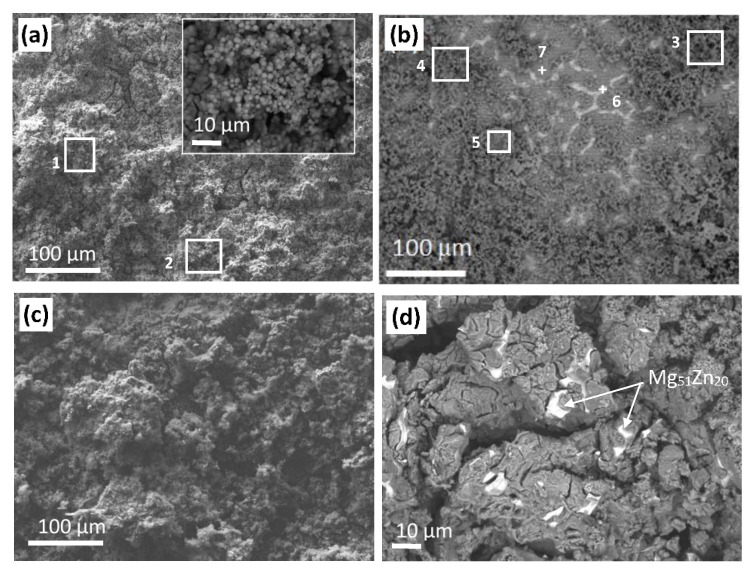
SEM images of (**a**,**b**) the Mg20Zn substrate (**c**,**d**) the Ca-P/chitosan_WG coating, after exposition for 110 h in the Hank’s solution at 37 °C.

Figure 7 shows the surface of the Mg20Zn substrate after immersion for 110 h in the Hank’s solution. Corrosion products are highly porous (image insert in Figure 7a). They cover a large part of the substrate surface, but not the entire surface (Figure 7a,b). The chemical composition of corrosion products was determined by means of EDS analysis in numerous sites (such as sites 1–7 in Figure 7a,b):8 ± 0.5 at.% carbon, 63.0 ± 2.0 at.% oxygen, 11.0 ± 0.9 at.% phosphorous, 8.5 ± 0.7 at.% magnesium, 8.0 ± 1.3 calcium, and 1.3 ± 0.1 at.% sodium. Magnesium comes from the substrate whereas all the other elements come from the Hank’s solution. Note that zinc, which is mainly encountered in the intermetallic phase, was not detected in the corrosion products. This suggests that the intermetallic phase is less oxidized than the matrix.

Surface observations in sites where the microstructure is still visible after immersion for 110 h in the Hank’s solution (Figure 7b,c) confirm that the matrix (α-Mg) is preferentially oxidized and covered by corrosion products. EDS analysis in site 6 (Figure 6b) corresponding to the intermetallic phase yields carbon 8.0 at.%, 38.0 at.% magnesium, 30.0 at.% oxygen, 17.6 at.% zinc, 4.0 at.% phosphorus, and 2.5 at.% calcium. The α-Mg matrix contains a higher amount of oxygen and elements from the solution: carbon, 45 at.% magnesium, 42.1 at.% oxygen, 6.1 at.% phosphorus, 4 at.% calcium, 1.1 at.% sodium, 1 at.% zinc, and 0.6 at.% chloride (Figure 6b, site 7).

The corrosion products formed on coated specimen (Ca/P_chitosan_WG) exhibit similar chemical composition such as these formed on the bare Mg20Zn alloy. They contain 10 ± 1.0 at.% carbon, 62.0 ± 1.0 at.% oxygen, 8.0 ± 2.0 at.% phosphorous, 11.5 ± 0.6 at.% magnesium, 6.0 ± 2.0 calcium, and 1.1 ± 0.2 at.% sodium. There is a difference in the chemical composition of the intermetallic phase Mg_51_Zn_20_ after the corrosion for uncoated and coated specimen. On the surface of the intermetallic phase, neither phosphorus nor calcium was found for the coated specimen.

**Figure 8:**
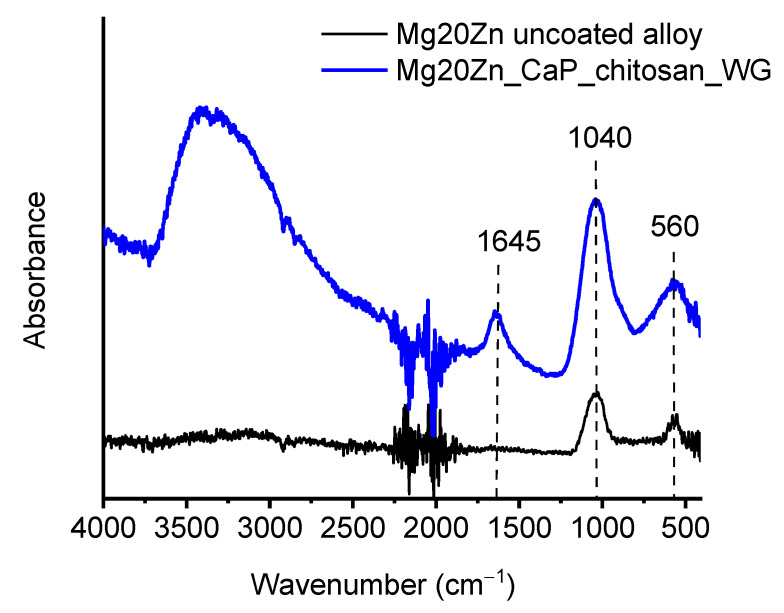
FTIR spectra of the Mg20Zn alloy (black curve) and the Ca-P/chitosan_WG coating (blue curve) after immersion in the Hank solution for 110 h. The relevant bands are described in the text.

Figure 8 shows the FTIR spectra of the Mg20Zn substrate and the Ca-P/chitosan_WG coating after immersion in the Hank’s solution for 110 h. The main absorption bands at 560 cm^−1^ and 1040 cm^−1^ are attributed to PO_4_^3−^, which confirms the formation of the calcium phosphate layer for both coated and uncoated specimens. In the spectrum obtained on the coated specimen (blue curve, Figure 8), the broad band in the range 3700–2500 cm^−1^ is due to overlapping of several bands, i.e., the stretching vibration of O-H, absorbed H_2_O, and N–H stretching of amine group, while the vibration bands of the C–H is at ~2920 cm^−1^ and ~2875 cm^−1^. The peak at ~1645 cm^−1^ can be assigned to the N–H bending mode of the amine group and bending mode of H_2_O. Band at ~1040 cm^−1^ and represent the C–O vibrations in the chitosan molecule [26,55,56].

## 4. Discussion

The X-ray photoelectron spectroscopy and FTIR measurements performed on the surface of specimens before and after corrosion tests delivered additional information concerning the structure and chemical composition of corrosion products. Therefore, the corrosion mechanism of magnesium alloys, coated by chitosan-based coatings, was proposed. In the Hank’s solution, the corrosion of magnesium alloys proceeds according with the following reactions:

Anodic reaction:

(5) $$\mathrm{Mg}\to {\mathrm{Mg}}^{2+}+2e$$

Cathodic reaction:

(6) $$2H_{2}O+2e\to H_{2}+2{\mathrm{OH}}^{-}$$

Overall reaction is expressed by Equation (1).

When the bare metal is exposed to solution or the coating is partly damaged, the anodic dissolution of magnesium and cathodic reduction of water molecules proceed according to Reactions (5) and (6). The presence of OH^−^ groups and magnesium cations cause the formation of main corrosion product Mg(OH)_2_ according to Reaction (1). In order to slow down the corrosion rate of magnesium alloy (Mg20Zn), Ca-P coating was deposited from the solution containing Ca(NO_3_)_2_ and NaH_2_PO_4_. The PO_4_^3−^ ions present in the solution react with calcium ions according to Reaction (7).

(7) $${\mathrm{Ca}}^{2+}+{\mathrm{PO}}_{4}^{3-}\to {\mathrm{Ca}}_{3}{\left({\mathrm{PO}}_{4}\right)}_{2}$$

Deposition of insoluble calcium phosphate blocked the penetration of aggressive chloride ions to the metal surface. Therefore, the calcium phosphate layer significantly hinders corrosion of the Mg20Zn substrate up to 40 h of immersion in the Hank’s solution, orange curve in Figure 6a. Deposition of the chitosan_WG coating on the bare Mg20Zn alloy decreases the corrosion rate of the substrate for 60 h of immersion in the Hank’s solution, blue curve in Figure 6a. The chitosan_WG solution is alkaline solution (pH = 11.5) and in the contact with the substrate containing magnesium, the following Reaction (8) proceeds.

(8) $$\mathrm{Mg}{\left(\mathrm{OH}\right)}_{2}+{\mathrm{Na}}_{2}{\mathrm{SiO}}_{3}={\mathrm{MgSiO}}_{3}+2\mathrm{NaOH}$$

Magnesium silicate is an insoluble salt, which is deposited on the substrate surface and slow down the corrosion rate of magnesium alloy. As it was revealed in Figure 6, the presence of two coatings on the surface of Mg20Zn alloy significantly decreases its corrosion rate in the Hank’s solution. Chitosan solution (chitosan_WG) containing water glass and Na_2_SiO_3_ is alkaline (pH = 11.5). Deposition of such chitosan_WG solution on the CaP coating causes the formation of insoluble calcium silicate according with the Reaction (9).

(9) $${\mathrm{Ca}}_{3}{\left({\mathrm{PO}}_{4}\right)}_{2}+3{\mathrm{Na}}_{2}{\mathrm{SiO}}_{3}=2{\mathrm{Na}}_{3}{\mathrm{PO}}_{4}+3{\mathrm{CaSiO}}_{3}$$

Formation of additional compound CaSiO_3_ insoluble in water causes the formation of a barrier layer and protects the alloy surface against corrosion. The solubility of CaSiO_3_ in water is three times lower than solubility of Ca_3_(PO_4_)_2_. Therefore, the synergistic effect due to the presence of the multilayer coating on the corrosion resistance of the Mg20Zn alloy is observed in the Hank’s solution. The highest corrosion resistance of Mg20Zn alloy, covered by the CaP/chitosan_WG coating, is caused by the formation of two compounds, CaSiO_3_ and Ca_3_(PO_4_)_2_, insoluble in water. The beneficial effect of the addition of CaSiO_3_ on the corrosion resistance of titanium alloy (Ti6Al4V) was presented in the paper [57].

Moreover, it should be emphasized that the chitosan is a pH sensitive polymer. In the acidic solution at pH 5 or lower, the chitosan molecules are protonated. However, in the solution at pH higher than 6, chitosan loses its positive charge and become insoluble. In this study, the corrosion test has been performed in the Hank’s solution at pH = 7.2. During the corrosion, the OH^−^ ions are produced and caused the increase of pH, therefore the chitosan exist as deprotonated molecules and cannot attract the negatively charged ions from the solution.

## 5. Conclusions

The structure of spin coated chitosan-based coatings deposited on the Mg20Zn alloy has been investigated. The corrosion resistance of Mg20Zn alloy covered by chitosan-based coatings has been studied in the Hank’s solution. The following conclusions can be drawn:

- The spin coating method allows to deposit uniform chitosan coatings on the magnesium alloy (Mg20Zn).
- The galvanic coupling between the α-Mg matrix and intermetallic phase Mg51Zn20 is a driving force for local corrosion. The corrosion proceeds preferentially in the α-Mg matrix.
- Obtained results show that, although rare microcracks are present in the coatings, the corrosion behavior of the alloy can be significantly improved. The next work will focus, in particular, on the optimization of the deposition process in order to remove these microcracks.
- The corrosion rate of Mg20Zn alloy covered by CaP_chitosan_WG coating and measured in the Hank’s solution is twice lower compared to bare alloy.
- The insoluble corrosion products (Ca_3_(PO_4_)_2_, CaSiO_3_) formed at the specimen surface hinder the penetration of chloride ions to metal surface.

## Data Availability

Data sharing not applicable.
